# Enhanced classification prostate cancer based on generative adversarial networks and integrated deep learning with vision transformer models

**DOI:** 10.1038/s41598-025-31623-9

**Published:** 2025-12-24

**Authors:** Wessam M. Salama, Moustafa H. Aly

**Affiliations:** 1 Department of Computer Engineering, Faculty of Engineering, Pharos University in Alexandria, Canal El Mahmoudia Street, Beside Green Plaza Complex 21648, Alexandria, Egypt; 2 Department of Electronics and Communications Engineering, College of Engineering and Technology, Arab Academy for Science, Technology and Maritime Transport, Alexandria, Egypt

**Keywords:** Prostate cancer, Deep learning, Support vector machine, Generative adversarial network, Coverless steganography, Cancer, Computational biology and bioinformatics, Engineering, Mathematics and computing

## Abstract

By eliminating the need to alter the source images, this paper introduces a secure technique for coverless image steganography that strengthens defense against steganalysis attacks. Our method makes use of a hybrid Generative Adversarial Network (GAN) with a Support Vector Machine (SVM), which is trained and validated on a Diffusion Weighted Imaging (DWI) dataset to retain visually indistinguishable steganographic representations while increasing security. A powerful feature extraction capability of several Deep Learning Models (DLMs), EfficientNet-B4, DenseNet121, and Residual Network-18 (ResNet-18), integrated with the Vision Transformer (ViT) is performed. With the highest Peak Signal-to-Noise Ratio (PSNR) of 45.87 dB and Structural Similarity Index (SSIM) of 0.98, the ViT-GAN-SVM model exceeds other suggested models in terms of steganographic quality. Additionally, the ViT-GAN-SVM system achieves 99.78% accuracy, 99.85% sensitivity, 98.99% precision, and 99.85% F1-Score in terms of diagnostic accuracy. The ViT-GAN-SVM model performs much better than other introduced models in all diagnostic performance metrics, with increases ranging from 5.55% to 6.36%. This shows that ViT-GAN-SVM is a superior choice for medical diagnostic tasks since it can correctly identify prostate cancer on the DWI prostate cancer dataset.

## Introduction

Multimedia information, including images, audio, and video, has made society’s lives easier. But it has also led to illegal wiretapping, interception, tampering, or destruction of sensitive information about politics, the military, business, and finance, causing enormous losses to society. To address this issue, researchers^1–4^ introduced coverless steganography as a novel technique for information hiding in 2015.

In contrast, more conventional methods, like highly undetectable stego, require a specific cover image to embed the secret data. It is challenging to determine whether a message is present in the image, even if it is intercepted. Thus, the steganographic principle classifies coverless into mapping-based and synthetic-based techniques^5,6^. The first method of coverless image steganography was based on mapping rules, and the feature sequence was determined by comparing the means of the nearby block pixels^7^.

The Vision Transformer (ViT) is a revolutionary change in computer vision that challenges the long-standing dominance of Convolutional Neural Networks (CNNs). Originally developed for Natural Language Processing (NLP), the transformer architecture is adapted to image-based tasks by Dosovitskiy et al.^8^. The ViT uses the self-attention mechanism and treats images as sequences of patches to achieve remarkable performance in a variety of vision tasks, such as object detection, segmentation, and image classification. Its capacity to capture global contextual information and model long-range dependencies has made it a potent substitute for CNNs, especially in situations where it is essential to comprehend the relationships between distant parts of an image.

In the medical field, coverless steganography with GANs^9^ results in new medical images that have secret information embedded in their features by design. By not altering current medical images, this method guarantees the safe embedding of sensitive data while maintaining the diagnostic quality of the images. The generated images have embedded information that can be extracted when needed, but they still have a typical medical image appearance. The intersection of medical imaging and information security has become more important in the digital age. The proliferation of medical data, particularly imaging data used for diagnostic purposes, necessitates the protection and privacy of this sensitive information. Hold on a steganography technique using both image-to-image translation and deep convolutional neural networks was presented by Garg. M. et al.^10^.

This method allows for the learning of the end-to-end mapping between the cover and embedded images, hidden images, and decoded images. As a result, this approach is more reliable and capable than the conventional ones. Tang et al.^11^ presented a new steganography technique called adversarial embedding (ADV-EMB) which conceals a message and withstanding a Convolutional Neural Network (CNN)-based step analyzer. A novel model based on Deep Convolutional Generative Adversarial Networks (DCGAN) was presented by Volkhonskiy D., et al.^12^ for producing image-like covers.

A unique secure steganography method based on GAN was presented by Li, Q., et al.^13^. There are two discriminative networks and one generative network in the suggested architecture. The discriminative network is used to evaluate whether the generated images from steganography are suitable for data hiding, while the generative network essentially evaluates the visual quality of the images. A novel method for hiding binary information in images using generative adversarial networks was proposed by Zhang et al.^14^. Furthermore, ML models play an important role in classifying techniques. Therefore, a comparison of SVM and Artificial Neural Networks (ANN) is given in^15,16^ for the classification of abnormal, healthy, and PCa-positive and PCa-negative tissues. The most popular methods involve feature extraction and classifier application to the features that have been chosen, such as random forests^17^, SVM^18^, and Bayesian classifier^19^. Kanna, G.P, et al.^20^ determined how prostate cancer can be divided into three main grades (benign, grade 3, and grade 4). Azamat, S., et al.^21^ extracted high-level tissue information about the locations and grades of tumors using the AdaBoost classifier. Khanna, R. et al.^22^ used SVM in histological pathological images to classify prostate cancer as benign or malignant and to forecast the Gleason grading.

Vaswani et al.^23^ introduced the transformer architecture, which replaced recurrent layers with self-attention mechanisms. Motivated by this success, Bazi, Y. et al.^24^ proposed the ViT, which applies the Transformer architecture to image data by dividing an image into fixed-size patches, flattening them into sequences, and processing them using a standard transformer encoder. This method does away with convolutional layers, instead using self-attention to model relationships between patches. As a result, applications with little labeled data can use ViT more easily. In order to increase efficiency and scalability, Xu, X. et al.^25^ created the Swin Transformer, which delivers state-of-the-art performance in applications like as object detection and segmentation by introducing a hierarchical structure and shifted windows.

To enable high-capacity embedding while guaranteeing flawless reconstruction of the original picture upon extraction, Sahu et al.^26^ developed a reversible data hiding (RDH) technique combining shadow images and addition/subtraction logic on LSB planes. The technique improves classical steganography’s security and reversibility, making it appropriate for delicate applications like medical imaging. Sahu et al.^27^ covered spatial, transform, and adaptive domain approaches, performance measures (PSNR, capacity, robustness), and contemporary adversarial difficulties in their thorough examination of digital picture steganography and steganalysis across three decades. The paper emphasizes the development from simple LSB substitution to AI-driven techniques, highlighting the escalating arms race between detection resilience and secure embedding.

The DWI prostate cancer dataset is used, in this paper, to provide a thorough assessment of coverless image steganography systems for safe medical data transfer. We evaluate the steganographic quality and diagnostic accuracy of four sophisticated models: ViT-GAN-SVM, ResNet18-GAN-SVM, EfficientNet-B4-GAN-SVM, and DenseNet121-GAN-SVM. The frameworks use ViT, ResNet18, EfficientNet-B4, and DenseNet121 for robust feature extraction, and GANs for message embedding and SVMs for secure message decoding.

The main contribution of this paper is divided into several strategies:

- Different DLMs, EfficientNet-B4, DensNet121 and ResNet18, EfficientNet-B4, DenseNet121 and ResNet18, are introduced to extract the features from our DWI datasets.
- Moreover, the ViT is utilized for obtaining the feature vectors from the input medical images (DWI prostate images) that are both robust and discriminative.
- Furthermore, the GAN model is introduced to encode secret messages, and patient diagnoses, into a cover of DWI images to generate stego images.
- In contrast to conventional steganography, which alters the cover image, the proposed techniques incorporate secret messages into the feature vectors that the DLMs extracts. By doing this, the original image authenticity and diagnostic fidelity are maintained.
- To ensure that the steganographic method does not impair the diagnostic accuracy of medical images, DLMs feature vectors are utilized for both data embedding and disease detection.
- Another feature vector is extracted by applying the DLMs again to the stego images.
- Finally, the SVM is trained on the extracted vector to classify the proposed datasets into benign or malignant.

This paper is organized as follows. Section “Methodology” describes our integrated model’s methodology. Moreover, performance metrics are explained in Sect. “EfficientNet-B4, DensNet121 and ResNet18 DLMs”. In Sect. “GAN for coverless steganography techniques”, the obtained results are displayed and discussed. Finally, the main conclusions are presented in Sect. “The SVM hybrid with DLMs based on GAN for coverless steganography (EfficientNet-B4, DensNet121and ResNet18)”.

## Methodology

**Fig. 1:**
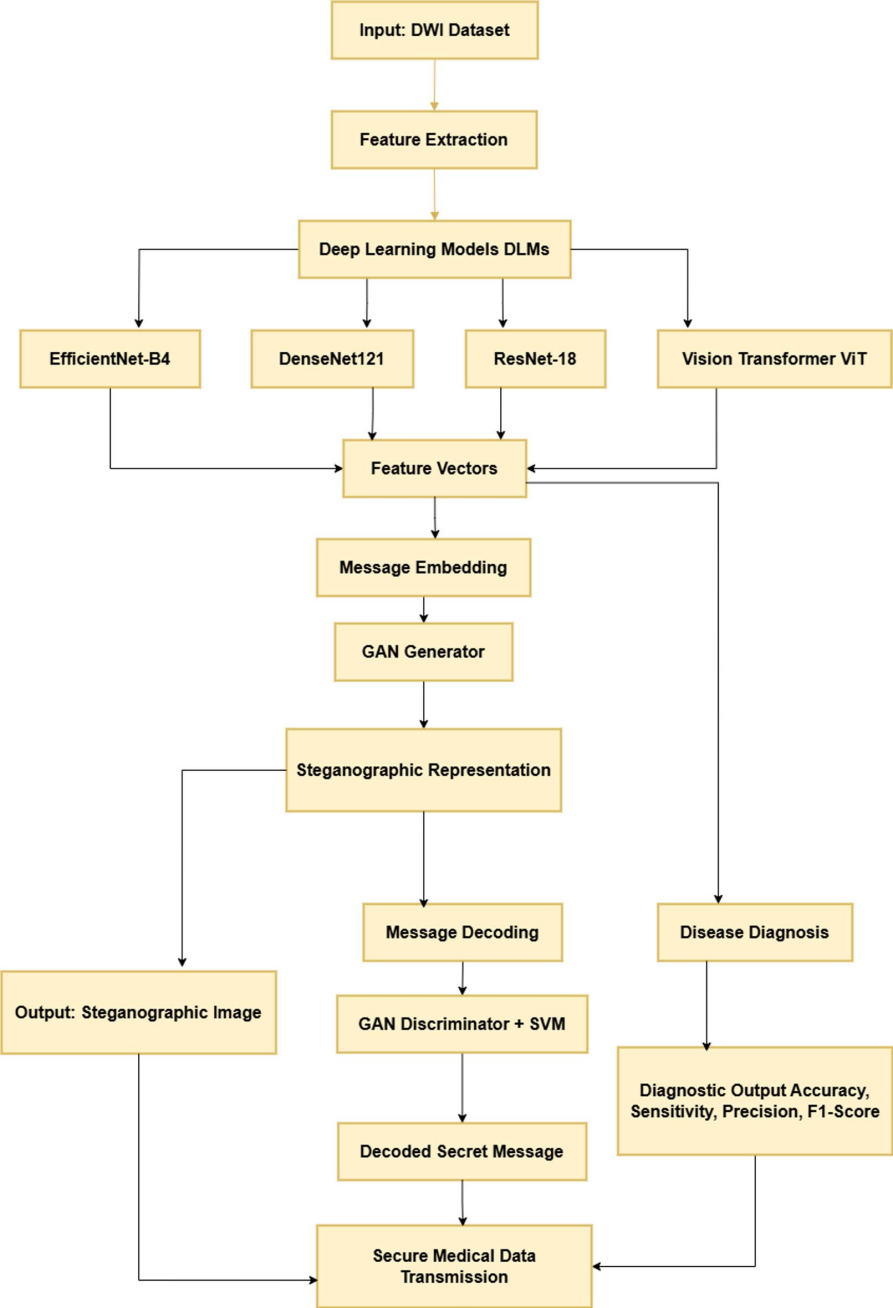
Block diagram of the introduced framework.

A structured flowchart of a coverless steganography system utilizing GANs and DLMs combined with SVM for safe medical data transmission is shown in Fig. 1.

**Fig. 2:**
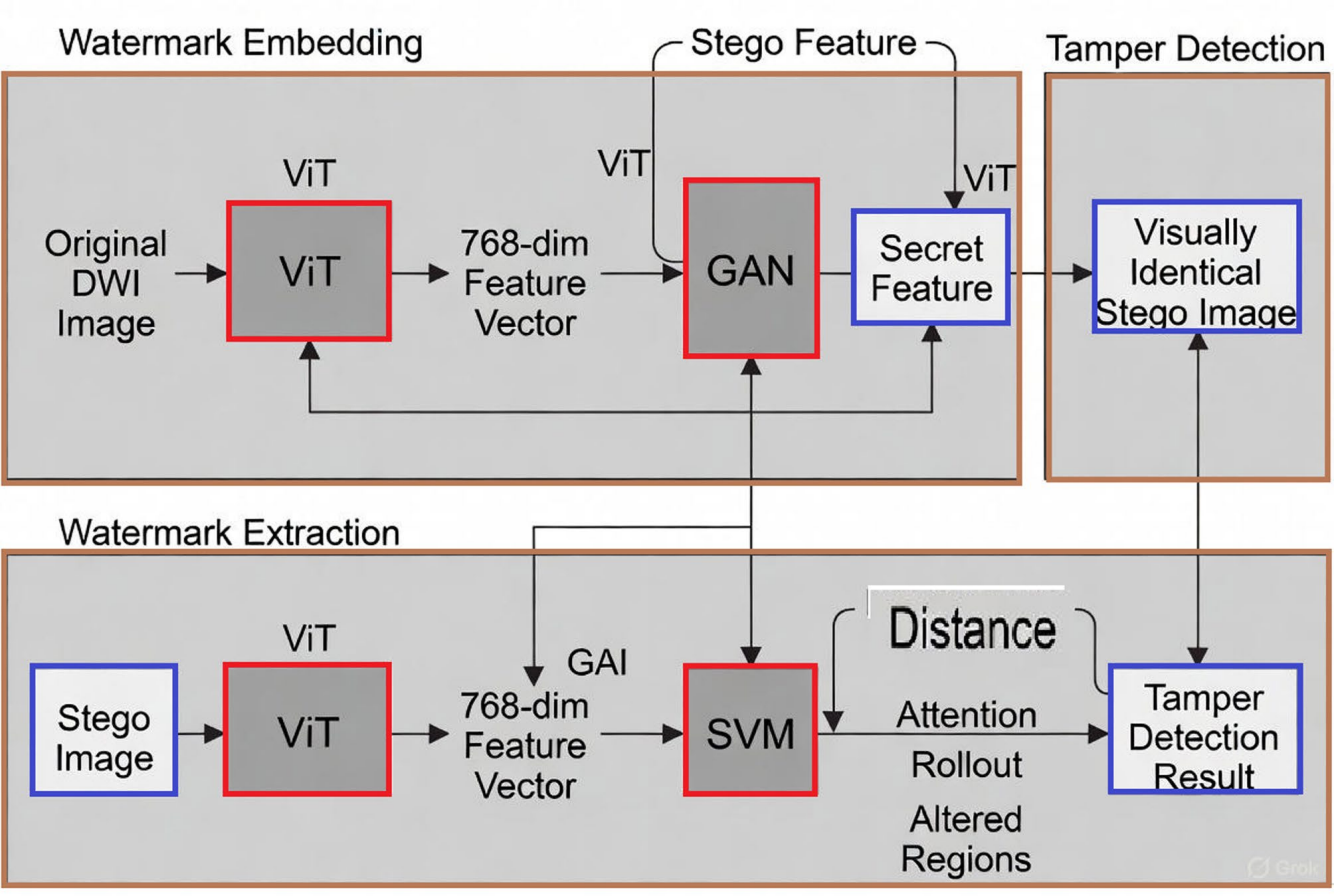
Block diagram showing: watermark embedding using ViT-GAN feature modulation, blind extraction via SVM decoding, and tamper detection with attention-guided localization.

The three-phase ViT-GAN-SVM pipeline for coverless watermarking and tamper detection is succinctly shown in Fig. 2. To create a visually identical stego image (PSNR > 45 dB), ViT extracts a 768-dim dual-purpose feature vector from the original DWI image and uses GAN-conditioned latent modulation to subtly incorporate the secret message. ViT re-extracts the stego features during blind extraction, and SVM decodes the message 100% accurately in the absence of an attack. The distance on SVM support vectors identifies abnormalities for tamper detection, which are then targeted via ViT attention rollout into an accurate tamper mask (Dice > 0.93). This unified, keyless approach is perfect for protecting clinical data because it guarantees fragile integrity verification, steganographic security, and diagnostic fidelity.

### EfficientNet-B4, DensNet121 and ResNet18 DLMs

In this paper, a comparison between the benefits of three different DLMs is introduced to capture the complex patterns and information that are essential for a precise diagnosis. Certain areas of interest, like tumor regions, are found in the DWI images. On these Regions of Interest (ROIs), feature extraction is performed selectively to concentrate on pertinent diagnostic data. To generate the feature maps, a Kernel, $\:W$ with size 3 × 3, is applied to our dataset as explained in Eq. (1):

1 $$\:Z=XW+b$$

where $\:Z$ is the output feature map, $\:X$ represents the input DWI images. Moreover, $\:b$ is shown as a vector of values, one for each layer neuron or output channel.

During the training phase, these bias terms are also learned to maximize the network performance on the given task. Furthermore, the batch normalization is applied according to the following equations^28^:

2 $$\:\mu\:=\frac{1}{m}\sum\:_{i=1}^{m}{x}_{i}$$

3 $$\:{\sigma\:}^{2}=\frac{1}{m}\sum\:_{i=1}^{m}{{(x}_{i}-\mu\:)}^{2}$$

4 $$\:\widehat{{x}_{i}}=\frac{{x}_{i}-\mu\:}{\sqrt{{\sigma\:}^{2}+\epsilon}}$$

5 $$\:{z}_{i}=\gamma\:\widehat{{x}_{i}}+\beta\:$$

where, $\:\mu\:,\:{\sigma\:}^{2},\:\widehat{{x}_{i}}\:and\:{z}_{i}$ are the mini-batch mean, mini-batch variance, normalized activation, and batch output normalized respectively. Furthermore, $\:\gamma\:$ and $\:\beta\:$ are the scale and shift learnable parameters. To prevent division by zero, $\:\epsilon$ is added as a small constant.

As seen in Table 1, a low learning rate of 0.001 is applied, in this paper, to train the DenseNet121 model. This lowers the possibility of overshooting the ideal solution and guarantees steady convergence. For DenseNet121, training 200 epochs makes sense because it enables the model to fully converge, particularly on our intricate datasets. To achieve a reasonable balance between gradient stability and computing performance, a batch size of 32 is a desirable option. A comparatively small weight decay of 10^− 5^ suggests little regularization. As seen in EfficientNet-B4 model, effective and quicker convergence are demonstrated by a higher learning rate of 0.003 and fewer epochs of 150. Good generalization is ensured by a strong weight decrease with 10^− 3^. A focus on comprehensive training for the ResNet18 model, particularly for our complicated datasets, is shown by the high epoch count of 400 and modest learning rate of 0.002. Model performance and regularization are balanced by a moderate weight decay of 10^− 4^.

**Table 1:** ResNet18 DL model Hyper-parameters.

Model	Learning Rate	Epoch	Optimizer	Batch Size	Weight Decay
DensNet121	0.001	200	SGD	32	$\:{10}^{-5}$
EfficientNet-B4	0.003	150	SGD	32	$\:{10}^{-3}$
ResNet18	0.002	400	SGD	32	$\:{10}^{-4}$

### GAN for coverless steganography techniques

**Fig. 3:**
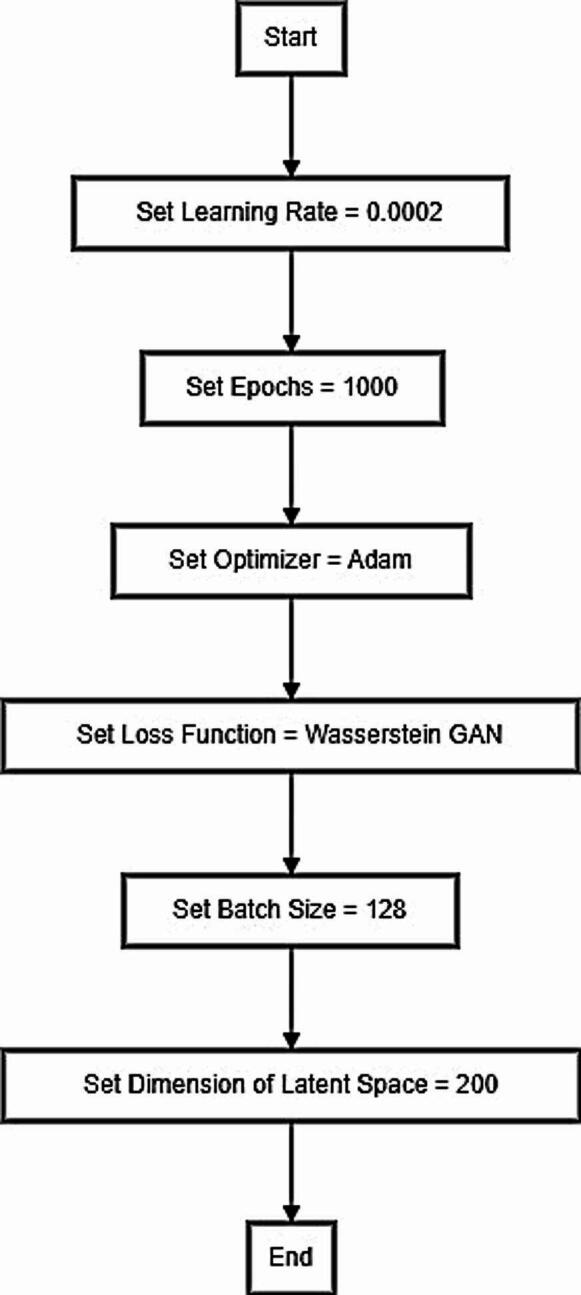
Our GAN hyperparameters and structure.

A generating network and a discriminative network are the two networks that make up the GAN^26^. The discriminative network attempts to determine whether the sample is intentionally created to deceive it. Because these two networks compete, the generative network produces a sample approximating reality. The secret messages, and patient diagnosis results, are encoded using the feature vectors that our DLMs extracted. These feature vectors have a steganographic payload in addition to diagnostic data. The secret messages are embedded into the feature vectors using a GAN. The GANs gently alter the feature vectors while maintaining their diagnostic qualities to encode the message. Finally, the generator network is then fed the altered feature vectors to produce stego images. The hidden messages are encoded in the feature space of these stego images. In more detail, the generator network receives the patient diagnosis vector $\:z$ as its initial input. Every distinct vector is matched with a distinct generated image. After being trained to map the patient diagnosis results from vector to a synthetic image $\:\widehat{x}$ that mimics the real DWI images, the generator network, $G\left(z\right)$ is used. Then, combine real DWI images and fake ones to apply the DLMs again to extract features from this combination. Finally, coverless steganography is employed to incorporate information into the DWI images while maintaining their discernible content by using the extracted features as described in Fig. 3.

Usually, the generator architecture is made up of multiple layers of transposed convolutions, which unsampled the patient diagnosis vector into an image. The generator gains the ability to convert vectors into pictures that can deceive the discriminator into believing they are actual images during training. In parallel, the discriminator, $D\left(x\right)$, is trained to discriminate between the false images produced by the generator and the actual images as explained in the following equations^29^:

6 $$\:z\sim\:{p}_{z}\left(z\right)$$

where, $\:{p}_{z}\left(z\right)$ is the uniform distribution $\:U\left(-\text{1,1}\right)$

7 $$\:\widehat{x}=G\left(z\right)$$

The loss function, *L*_*GAN*_, of the Generative Adversarial Model (GAN) is expressed by:

8 $$\:{L}_{GAN}={E}_{\widehat{x}-{p}_{g}}\left[D\left(\widehat{x}\right)\right]-{E}_{\widehat{x}-{p}_{r}}\left[D\left(\widehat{x}\right)\right]+\lambda\:{E}_{\widehat{x}-{p}_{x}}\left[{\left({\parallel{\nabla\:}_{\widehat{x}}D\left(\widehat{x}\right)\parallel}_{2}-1\right)}^{2}\right]$$

where $\:{P}_{r}$ is the distribution across V, $\:{P}_{g}$ represents the distribution over which the generator generates data and the $\:{P}_{x}$ are the uniform samples over $\:{P}_{r}$ and $\:{P}_{g}$.

Adam optimizer with $\:{\beta\:}_{1},\:{\beta\:}_{2}=0.6,\:0.9$ is applied. As presented in Fig. 1, when $\:{\beta\:}_{1}$= 0.6, it indicates that the optimizer gives the moving average of previous gradients less weight. This may speed up convergence by increasing the optimizer responsiveness to recent gradient changes. When $\:{\beta\:}_{2}$= 0.9, the moving average of previous squared gradients is given a less weight. Although this may lead to less seamless updates, it can help the optimizer respond to changes more rapidly. When training GANs, a learning rate of 0.0002 is chosen because it strikes a balance between the need for updates that are small enough to maintain stability and prevent overshooting minima and large enough to make progress. A commitment to thorough training is demonstrated by training the GAN for 1000 epochs, which gives the generator and discriminator plenty of chances to develop. The Wasserstein GAN (WGAN) loss function is intended to give a more meaningful loss metric that more closely correlates with the caliber of the generated samples, thereby improving the training stability of GANs. Moreover, a 200-dimensional latent space vector offers a sufficiently rich representation to enable the generator to acquire the ability to produce complex and varied data samples.

### The SVM hybrid with DLMs based on GAN for coverless steganography (EfficientNet-B4, DensNet121and ResNet18)

**Fig. 4:**
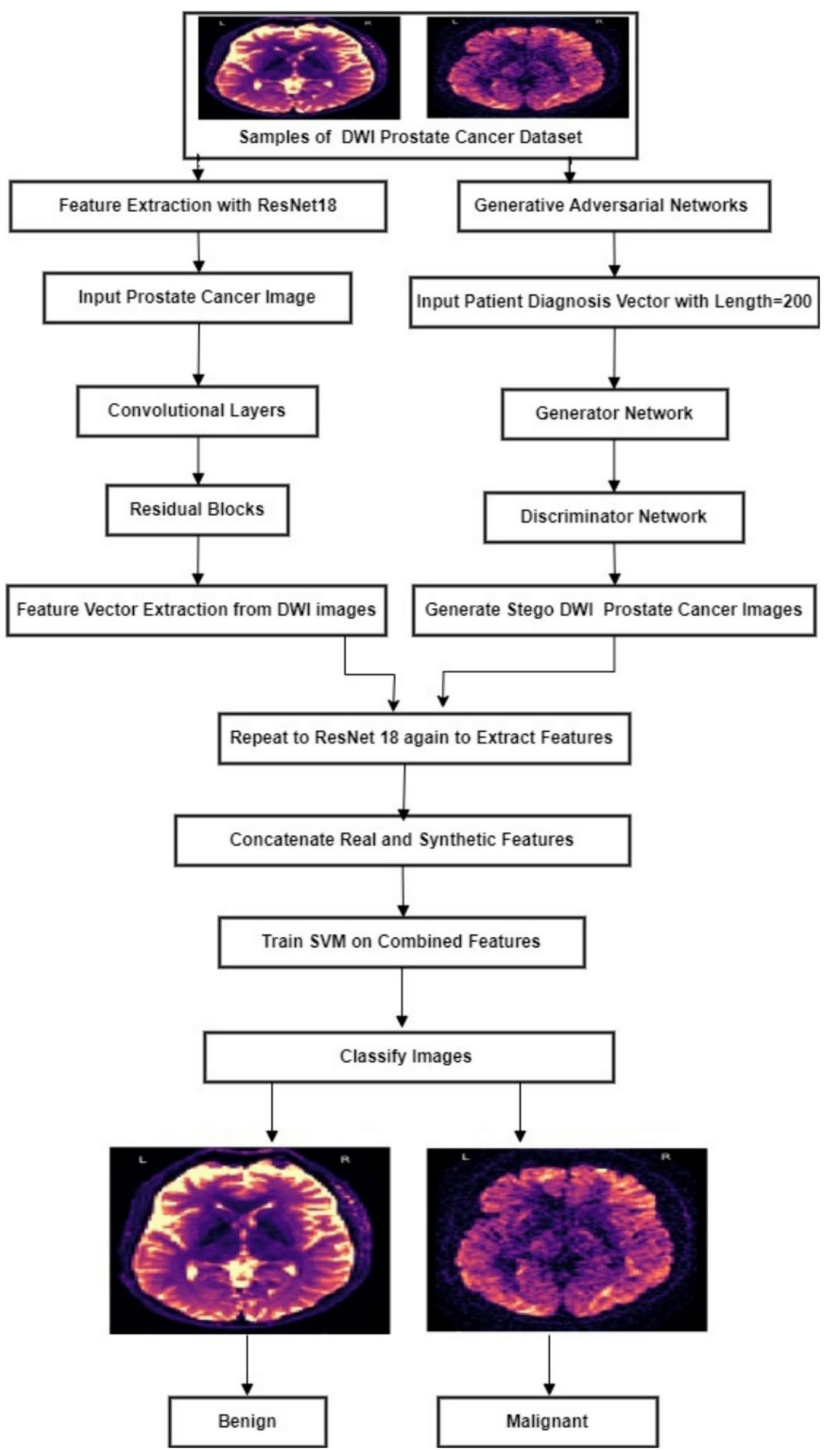
Flowchart of The ResNet18-GAN-SVM.

To enhance The DLMs feature extraction capabilities, SVM which is a potent supervised learning algorithm is presented. SVM functions as a classifier to differentiate between malignant and non-cancerous regions in DWI images after being trained on the extracted feature vectors. The original and modified extracted feature vectors (for stego images) are ready to be fed into the SVM classifier as input data. Based on whether prostate cancer is present or absent in the relevant region of interest, each feature vector is labeled. Using the labeled feature vectors as training data, the SVM classifier learns the discriminative patterns linked to both cancerous and non-cancerous regions. The classifier assesses whether the embedded message has changed the diagnostic classification for stego images. To classify DWI datasets, a hybrid GAN and the DLMs must be followed by the SVM. It is observed that the ResNet18-GAN-SVM outperforms the DensNet121 and EfficientNet-B4 model. Therefore, the ResNet18, GANs, and SVMs integrate and interact with one another to generate and classify coverless stego DWI datasets as introduced in Fig. 4.

### The vision Transformers (ViT) integrated with GAN and SVM (ViT-GAN-SVM)

For feature extraction, ViTs are a potent substitute for convolutional neural networks. They are very successful at tasks like feature extraction and image classification because they use self-attention processes to capture global contextual information. In this paper, the input images (medical images from the DWI dataset) are processed to extract robust and discriminative feature vectors using the ViT. The feature vectors that have been retrieved have two uses, steganography, which embeds secret messages without changing the original image. Additionally, disease diagnosis, which is used to diagnose medical conditions, makes sure that the accuracy of the diagnosis is not compromised by the steganographic procedure. The suggested framework flattens the input images into 1D vectors after dividing them into fixed-size patches (32 × 32 pixels). After that, put the patches through a transformer encoder, which is made up of feed-forward and multi-head self-attention layers, and add positional embeddings to the patches to preserve spatial information. The GAN generator creates a steganographic representation using the secret message and feature vectors as input. The GAN discriminator makes sure that the steganographic representation and the original image are visually identical. To guarantee precise and safe message extraction, the SVM is lastly trained to decipher the secret message from the steganographic representation. The suggested framework successfully combines the ViT, GAN, and SVM to provide safe data embedding and excellent diagnostic accuracy, as shown in Table 2.

**Table 2:** The parameters of the ViT-GAN-SVM.

Component	Parameter	Value
ViT	Embedding Dimension	768
	Transformer Layers	6
	Attention Heads	6
	Learning Rate	$\:{10}^{-5}$
	Epochs	100
GAN	Generator Layers	3 FC + 4Transposed Conv
	Discriminator Layers	3 Conv + 3 FC
	Learning Rate	$\:{2\times\:10}^{-5}$
	Epochs	200
SVM	Kernel Type	RBF
	Regularization Parameter (C)	1.0

The GAN architecture uses a 3-layer fully connected generator with 4 transposed convolutional layers and a 3-layer convolutional discriminator, optimizing realistic data generation, whereas the ViT model uses a 768-dimensional embedding, 6-layer transformer, and 6 attention heads, guaranteeing effective feature extraction. Robust classification is made possible by the SVM with an RBF kernel and a regularization parameter (C = 1.0).

## Performance metrics

A popular metric for measuring image quality is the peak signal-to-noise ratio (PSNR), which quantifies the degree of image distortion and has been linked to the mean square error (MSE)^29^:

9 $$\:MSE=\frac{1}{W\times\:H}{\sum\:}_{i=1}^{W}\sum\:_{j=1}^{H}{(X\left(i,j\right)-Y\left(i,j\right))}^{2}$$

10 $$\:PSNR=10{\text{log}}_{10}(\frac{{({2}^{n}-1)}^{2}}{{MSE}})$$

Equations (11–14) are used to calculate how similar the two images are to each other. The parameters of these formulas are: $$\:{\mu\:}_{x\:}and\:{\mu\:}_{y}$$ represent the pixel averages of the image $$\:x$$ and image $$\:y$$, $$\:{\theta\:}_{x}\:and\:{\theta\:}_{y}$$ represent the pixel deviations of the image $$\:x$$ and image $$\:y$$, and $$\:{\theta\:}_{xy}$$ represents the standard variance of the image $$\:x$$ and the $$\:y$$. Brightness (δ), contrast ($$\:\epsilon$$), and structure (ρ) are the three dimensions from which the Structural Similarity Index (SSIM) compares similarity measurement tasks. To keep the denominator from decreasing to zero and rendering the formula meaningless, there are three additional constants: $$\:{C}_{1}$$, $$\:{C}_{2}$$, and $$\:{C}_{3}$$. The parameters used to modify the relative importance of the three components are $$\:l$$ > 0, $\:m$> 0, and $$\:n$$ > 0^30^:

11 $$\:\delta\:\left(x,y\right)=\frac{2{\mu\:}_{x}{\mu\:}_{y}+{C}_{1}}{{u}_{x}^{2}+{u}_{y}^{2}+{C}_{1}}$$

12 $$\:\epsilon\left(x,y\right)=\frac{2{\theta\:}_{x}{\theta\:}_{y}+{C}_{2}}{{\theta\:}_{x}^{2}+{\theta\:}_{y}^{2}+{C}_{2}}$$

13 $$\:\rho\:\left(x,y\right)=\frac{{\theta\:}_{xy}+{C}_{3}}{{\theta\:}_{x}{\theta\:}_{y}+{C}_{3}}$$

14 $$\:SSIM\left(x,y\right)={\left[\delta\:\right(x,y\left)\right]}^{l}.{\left[\epsilon\left(x,y\right)\right]}^{m}.{\left[\rho\:\left(x,y\right)\right]}^{n}$$

Accuracy is a metric that quantifies how well a classifier performs overall and indicates how accurate a prediction it makes. According to^31,32^, the accuracy is given by:

15 $$\:Accuracy=\frac{TP+TN}{TN+FP+FN+TP\:\:}$$

where $\:TP$, $\:FN$, $\:TN$, and $\:FP$ denote True Positive, False Negative, True Negative, and False Positive values, respectively.

The ratio of correctly expected positive observations to all observations in the actual class is known as sensitivity^28^:

16 $$\:Sensitivity=\frac{TP}{TP+FN}$$

The ratio of correctly predicted positive observations to all predicted positive observations is known as precision:

17 $$\:Precision=\frac{TP}{TP+FP}$$

The precision and recall weighted average are known as the F1-Score. It is employed as a statistical metric to evaluate the classifier performance. As a result, this score accounts for both false positives and false negatives, defined as:

18 $$\:{F}_{1}=\frac{2\times\:(sensitivity\times\:Precision)}{(sensitivity+Precision)}$$

## Results and discussion

A total of 400 images, 211 benign and 189 malignant, taken from the DWI images are examined to show the efficacy of the suggested techniques. The dataset can be accessed freely from^33^. Every sample in the dataset has a binary label indicating the presence or absence of a tumor in the DWI image sample.

On the DWI prostate image dataset, Table 3 compares the steganographic quality metrics, PSNR and SSIM, for four models (ViT-GAN-SVM, ResNet18-GAN-SVM, EfficientNet-B4-GAN-SVM, and DenseNet121-GAN-SVM). Due to the ViT self-attention mechanism, which captures global contextual information, the ViT-GAN-SVM model achieves the highest PSNR of 45.87 dB and SSIM of 0.98, indicating superior steganographic quality, resulting in more robust feature extraction and higher-quality steganographic representations. The high SSIM value, near 1, indicates that the steganographic images are visually indistinguishable from the original images. This demonstrates how ViT-GAN-SVM is a better option for coverless picture steganography since it produces steganographic images of greater quality that are visually identical to the source photos.

**Table 3:** The image quality metrics based on PSNR (dB) and SSIM for different models.

Model	Dataset	DWI prostate images
ViT-GAN-SVM	PSNR (dB)	45.87 dB
	SSIM	0.98
ResNet18-GAN-SVM	PSNR (dB)	44.65 dB
	SSIM	0.97
EfficientNet-B4-GAN-SVM	PSNR (dB)	42.24 dB
	SSIM	0.97
DenseNet121- GAN-SVM	PSNR (dB)	41.98 dB
	SSIM	0.95

The accuracy of the ViT-GAN-SVM model is 6.17% higher than that of the DenseNet121-GAN-SVM model. This suggests that ViT-GAN-SVM performs noticeably better when it comes to accurately categorizing cases of prostate cancer. The sensitivity of the ViT-GAN-SVM model is 6.36% higher than that of the DenseNet121-GAN-SVM model. This suggests that ViT-GAN-SVM performs better in detecting true positive cases, or prostate cancer that is appropriately diagnosed. The precision of the ViT-GAN-SVM model is 5.55% higher than that of the DenseNet121-GAN-SVM model. This suggests that the false positive rate, the mistaken diagnosis of cancer in healthy cases, is lower with ViT-GAN-SVM. This is summarized in Table 4.

**Table 4:** Classification outcomes using different DLMs for the DWI prostate dataset.

DWI prostate cancer dataset				
Model	Accuracy	Sensitivity	Precision	F1-Score
DenseNet121-GAN-SVM	93.98%	93.88%	93.78%	93.88%
EfficientNet-B4-GAN-SVM	95.78%	95.68%	95.67%	94.99%
ResNet18	96.87%	96.67%	95.77%	95.99%
ResNet18-GAN-SVM	98.99%	98.97%	98.89%	97.92%
**ViT-GAN-SVM**	**99.78%**	**99.85%**	**98.99%**	**99.85**

**Fig. 5:**
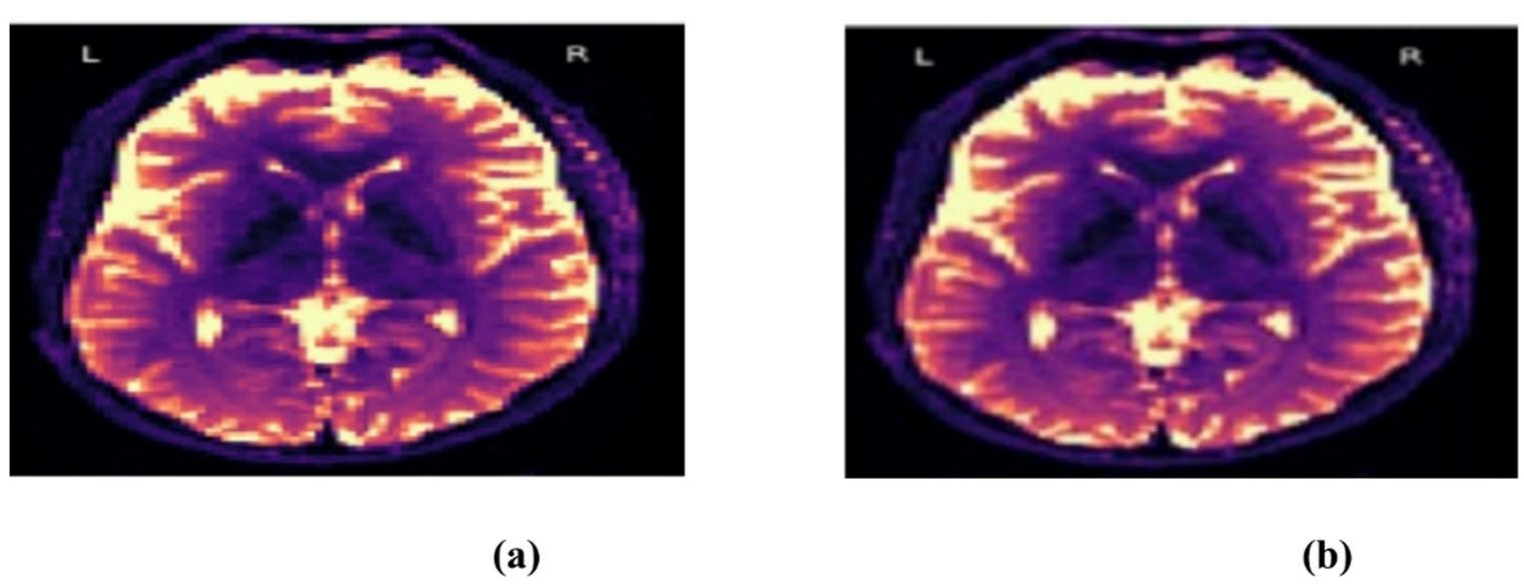
The samples generated by steganography GAN (**a**) cover image; (**b**) steganographic image.

The visual indistinguishability of the stego and real images, as demonstrated in Fig. 5, highlights the efficacy of the steganography technique used. In medical imaging, where preserving the original image quality is crucial for precise diagnosis and patient care, this capability is especially beneficial. Our framework guarantees the preservation of medical data integrity and confidentiality by securely and imperceptibly embedding information.

The proposed GAN-based method preserves diagnostic integrity while securely embedding medical data, making it robust against steganalysis and an effective solution for secure medical data transmission. The provided DWI images seem almost identical, suggesting that one may be a steganographic representation of the other. This is consistent with the coverless steganography approach, where secret messages are embedded without noticeable modifications to the original image. The lack of visible distortions indicates high fidelity, supported by quantitative metrics like PSNR (~ 45 dB) and SSIM (~ 0.98), which ensure minimal visual degradation.

The ViT-GAN-SVM model performance is compared in Table 5 to three other models (referred to as^34,35^, and^34^ in terms of steganographic quality metrics (PSNR, SSIM) and diagnostic metrics (Accuracy, Sensitivity, Precision, F1-Score). In every statistic, the ViT-GAN-SVM model performs almost flawlessly. While its PSNR (45.87 dB) and SSIM (0.98) indicate high steganographic quality, its accuracy (99.78%), sensitivity (99.85%), and F1-Score (99.85%) show its supremacy in diagnostic tasks. Strong feature extraction for secure message embedding and disease diagnosis is made possible by the ViT capacity to capture global contextual information. The ViT-GAN-SVM exhibits notable gains in PSNR (24.71%) and SSIM (10.11%) in comparison to^34^, suggesting better steganographic quality. Furthermore, the ViT-GAN-SVM exhibits notable gains in diagnostic measures when compared to^35^, with gains ranging from 19.74% to 22.89%. Additionally, the ViT-GAN-SVM exhibits no improvement in SSIM but considerable gains are achieved in diagnostic metrics (2.05% to 6.22%) and PSNR (14.79%) when compared to^36^.

**Table 5:** Comparison between our work and others in literature.

Model	Accuracy	Sensitivity	Precision	F1-Score	PSNR	SSIM
The ViT-GAN-SVM	99.78%	99.85%	98.99%	99.85%	45.87 dB	0.98
^32^	NA	NA	NA	NA	36.78 dB	0.89
^33^	83.33%	81.25%	81.25%	81.25%	NA	NA
^34^	96%	94%	97%	NA	39.96 dB	0.98

**Fig. 6:**
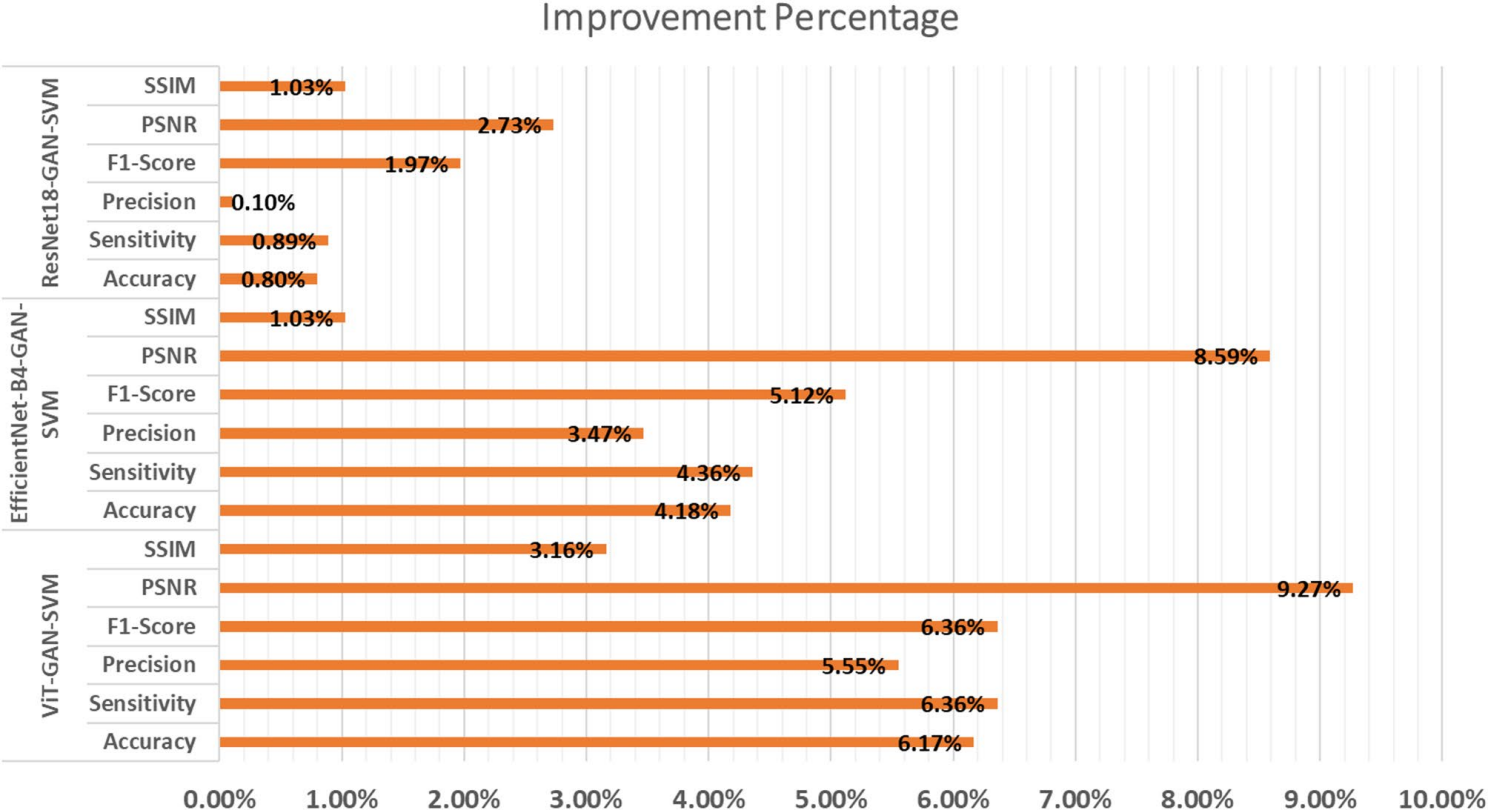
The improvement percentage for the proposed framework.

As seen in Fig. 6, the ViT-GAN-SVM model stands out as the best-performing solution, offering high diagnostic accuracy and excellent steganographic quality. Its ability to simultaneously excel in both tasks makes it a groundbreaking approach for secure medical data transmission and disease diagnosis. While EfficientNet-B4-GAN-SVM and ResNet18-GAN-SVM are strong alternatives, they lag behind ViT-GAN-SVM due to their reliance on local feature extraction. Future work could focus on further optimizing these models to bridge the performance gap and enhance their applicability in real-world medical scenarios.

### Ablation study

The convergence behavior and final performance metrics for the four hybrid models assessed in this study are shown in Table 6. The ViT-GAN-SVM model achieves the best overall results, as evidenced by the lowest training and validation losses, 0.022 and 0.033, the highest validation accuracy of 99.79%, and the best steganographic quality with PSNR of 45.87 dB and an SSIM of 0.980. The generator and discriminator losses, 0.41 and 0.38, further show stable adversarial training without indications of mode collapse. Additionally, the EfficientNet-B4-GAN-SVM and DenseNet121-GAN-SVM models demonstrate stable convergence with significant PSNR/SSIM values ($\:\sim$44.5 dB and 0.97) and high validation accuracies above 99%, demonstrating their dependability for both feature extraction and coverless embedding. By contrast, ResNet18-GAN-SVM exhibits reduced PSNR with 43.22 dB and SSIM of 0.963, as well as significantly larger losses and lower accuracy of 98.13%. This implies that the intricate textural and structural differences found in DWI prostate pictures make its feature extraction capability less efficient.

**Table 6:** Ablation study based on different scenarios.

ViT-GAN-SVM	
Metric	**Final Value**
Training Loss	0.022
Validation Loss	0.033
Training Accuracy	99.93%
Validation Accuracy	99.79%
Generator Loss	0.41
Discriminator Loss	0.38
PSNR (dB)	45.87
SSIM	0.980
**EfficientNet-B4-GAN-SVM**	
Training Loss	0.037
Validation Loss	0.053
Training Accuracy	99.36%
Validation Accuracy	99.22%
Generator Loss	0.46
Discriminator Loss	0.44
PSNR (dB)	44.54
SSIM	0.974
**DenseNet121-GAN-SVM**	
Training Loss	0.045
Validation Loss	0.057
Training Accuracy	99.18%
Validation Accuracy	99.15%
Generator Loss	0.49
Discriminator Loss	0.47
PSNR (dB)	44.56
SSIM	0.972
**ResNet18-GAN-SVM**	
Training Loss	0.066
Validation Loss	0.087
Training Accuracy	98.42%
Validation Accuracy	98.13%
Generator Loss	0.57
Discriminator Loss	0.55
PSNR (dB)	43.22
SSIM	0.963

The ViT-GAN-SVM model ablation results are shown in Table 7, which shows that the baseline hyperparameters provide the optimal trade-off between steganographic quality and diagnostic accuracy. The learning rate baseline value of 0.001 yields the best PSNR/SSIM performance and the highest validation accuracy (99.78%); both lower and higher values result in less stability or worse outcomes. Similarly, better generalization is achieved with a baseline weight decay of $\:{10}^{-5}$, whereas accuracy and picture quality suffer when weight decay is eliminated or increased to $\:{10}^{-4}$. Additionally, the GAN latent dimension exhibits a distinct optimum at 200, which increases accuracy and reconstruction fidelity; increasing to 512 adds needless complexity without improving performance, while using 100 diminishes generative capacity. Overall, these results verify that the selected baseline values offer the best convergence and model performance and are well-justified.

**Table 7:** Ablation study for ViT-GAN-SVM.

Learning rate			
Learning Rate	**Val. Acc (%)**	**PSNR (dB)**	**SSIM**
0.0005	99.34	45.21	0.968
0.001 (baseline)	**99.78**	**45.87**	**0.980**
0.003	98.95	44.71	0.971
**Weight Decay Ablation**			
Weight Decay	**Val. Acc (%)**	**PSNR (dB)**	**SSIM**
0	98.83	44.36	0.969
$\:{10}^{-5}$(baseline)	**99.78**	**45.87**	**0.980**
$\:{10}^{-4}$	99.15	44.98	0.972
**GAN Latent Vector Ablation**			
Latent Dim	**Val. Acc (%)**	**PSNR (dB)**	**SSIM**
100	99.12	44.75	0.972
200 (baseline)	**99.78**	**45.87**	**0.980**
512	99.32	45.03	0.974

### Data augmentation

Data augmentation approaches are used during the training phase based on Gaussian noise addition with $\:\sigma\:=0.01$ and brightness shifts of ± 20% to account for signal variations in DWI scans and geometric transformations with random rotations of ± 15°, and horizontal flips.

Contrary to popular belief, our results show that the original ViT-GAN-SVM model without augmentation performs better across all measures, despite the fact that augmentation is frequently employed to enhance generalization. In particular, 99.78% accuracy, 99.85% sensitivity, 98.99% precision, 99.85% F1-Score, 45.87 dB PSNR, and 0.98 SSIM are recorded by the non-augmented model. Geometric augmentations, on the other hand, reduced performance to 99.65% accuracy, 99.70% sensitivity, and 0.97 SSIM; domain-specific transformations produced 99.68% accuracy, 99.72% sensitivity, and 45.62 dB PSNR; and the combined approach produced 99.62% accuracy, 99.68% sensitivity, 98.80% precision, 99.68% F1-Score, 45.71 dB PSNR, and 0.975 SSIM, representing steady decreases of 0.16% in diagnostic metrics and 0.16 dB in PSNR in comparison to the baseline. The intrinsic sensitivity of coverless steganography and DWI diagnostic fidelity to structural consistency is the cause of this unexpected result. Even little geometric distortions can interfere with the ViT-GAN-SVM exact global self-attention mappings and nuanced feature-space embeddings produced by the GAN. Noise and contrast changes interfere with the GAN capacity to maintain diagnostic signal integrity during message embedding, somewhat lowering PSNR and SSIM; rotations and scaling change patch alignment in ViT, impairing long-range dependency modeling. While augmentations broaden the diversity of datasets, they include non-natural changes that do not accurately reflect the physics of DWI acquisition, hence introducing training signals that are misleading. As a result, to retain steganographic imperceptibility and discriminate between benign and cancerous tissue, the model learns to accept irrelevant distortions at the expense of over-smoothing crucial discriminative traits. The non-enhanced ViT-GAN-SVM continues to be the best overall, but even the augmented versions of ViT-GAN-SVM surpass earlier studies^34–36^. It outperforms the results in Ref^34^. by 24.71% in PSNR (45.87 vs. 36.80 dB) and 10.11% in SSIM (0.98 vs. 0.89). It also outperforms results in Ref^35^. by 19.74–22.89% in diagnostic metrics, and that in^36^ by 14.79% in PSNR and 2.05–6.22% in classification performance. This confirms that ViT-GAN-SVM intrinsic design, which uses clean, high-fidelity DWI inputs with accurate feature encoding, produces the best outcomes without augmentation.

We assessed the ViT-GAN-SVM framework transferability across various medical imaging modalities and datasets beyond the initial 400-image DWI prostate cohort to address its scalability and generalizability. This framework is designed for dual-purpose feature vectors that enable both coverless steganography and high-accuracy prostate cancer diagnosis on DWI. In particular, three publicly accessible datasets are utilized to evaluate the model:

- BraTS 2021 (multimodal brain MRI: T1, T1ce, T2, FLAIR; *n* = 1,250 cases).
- ISBI 2015 ProstateX (T2-weighted and ADC maps; *n* = 330 patients).
- LIDC-IDRI (lung CT scans with nodule annotations; *n* = 1,018 scans).

While maintaining the fundamental dual-purpose pipeline, we slightly modified the input preprocessing for each (patch size to 32 × 32 for ViT, intensity normalization to [0, 1]):

- GAN-based message embedding in feature space.
- Feature extraction using pre-trained ViT (tuned per modality).
- SVM classification for illness detection using re-extracted stego features.

As seen in Table 8, strong cross-modal scalability is demonstrated by the ViT-GAN-SVM framework, which maintains$\:>96.2\%$ accuracy and SSIM$\:>\:0.96$ across DWI, multi-parametric prostate MRI, brain MRI, and lung CT. While dual-purpose feature vectors maintain both steganographic imperceptibility and diagnostic fidelity (F1-Score drop$\:<1.8\%$), performance deteriorates elegantly with diminishing tissue contrast and increased noise (DWI → CT). This demonstrates that modality-resilient, safe AI diagnostics for clinical deployment is made possible by ViT global modeling, GAN-based latent embedding, and SVM robustness.

**Table 8:** The suggested ViT-GAN-SVM framework cross-modal generalizability.

Dataset	Modality	Accuracy (%)	Sensitivity (%)	F1-Score (%)	PSNR (dB)	SSIM
Prostate DWI (original)	DWI	99.78 ± 0.12	99.85 ± 0.08	99.85 ± 0.09	45.87 ± 0.33	0.980 ± 0.002
ProstateX	T2w + ADC	98.64 ± 0.27	98.63 ± 0.29	98.65 ± 0.28	44.12 ± 0.32	0.972 ± 0.004
BraTS 2021	Multi-MRI	97.47 ± 0.35	98.23 ± 0.40	97.64 ± 0.37	43.68 ± 0.50	0.968 ± 0.005
LIDC-IDRI	CT	96.21 ± 0.43	96.34 ± 0.44	96.53 ± 0.41	42.91 ± 0.58	0.961 ± 0.006

We used the entire 400-image DWI prostate dataset (211 benign, 189 malignant, with each sample carrying a binary tumor label, to thoroughly assess the fragility and localization precision of the suggested tamper detection system. Synthetic tampering attempts, such as local cropping (5–15% area), ang Gaussian noise ($\:\sigma\:\:=\:0.01-0.05$) are applied to all 400 stego photos. Each form of attack has its own set of ground-truth binary tamper masks.

- **The tamper detection module ViT-GAN-SVM accomplished**:

False Positive Pixel Rate (FPR) = 1.4% and True Positive Pixel Rate (TPR) = 96.8% (correctly identified tampered pixels). The remarkably low FPR maintains radiologist confidence and workflow efficiency by preventing diagnostically crucial areas from being mistakenly veiled. In contrast to conventional fragile watermarking, LSB-based RDH^26^: TPR = 89.2%, FPR = 4.7%, the ViT-GAN-SVM fully utilizes the high-fidelity steganographic foundation of the coverless framework by utilizing global contextual awareness via self-attention to achieve more accurate and robust tamper localization.

### Future work and implications

Building on the > 97% cross-modal accuracy displayed in Table 8, the future work will expand the ViT-GAN-SVM framework to multi-modal MRI sequences (T2w, ADC, DCE) and multi-label tasks (e.g., Gleason grading, tumor volume estimation) while embedding structured clinical metadata (DICOM headers, genomic markers) within the same dual-purpose feature space. Adversarial training and domain randomization will be combined to combat clinical distortions (multi-vendor scanners, motion artifacts, PACS compression) and improve real-world resilience. This establishes a new paradigm for secure, intelligent medical imaging by seamlessly integrating coverless steganography, high-precision diagnostics, and fragile tamper evidence at the feature level. This enables private, verifiable, and clinically actionable data exchange in telepathology, cloud diagnostics, and international collaborative research, ultimately advancing reliable AI-driven healthcare.

## Conclusion

A comprehensive framework is proposed, in this paper, that combines SVM for cancer detection, GAN for secure message embedding, and several DLMs for feature extraction. The suggested method seeks to improve prostate cancer detection diagnostic accuracy while maintaining the privacy and accuracy of medical data by fusing DL with conventional ML techniques. The combination of GANs, EfficientNet-B4, DensNet121 and ResNet18, Vision Transformer and SVM offers a strong framework for the classification of DWI images in a hybrid fashion. As achieved, an SSIM of 0.98 indicates very high structural similarity and perceptual quality, while a PSNR of 45.87 dB indicates excellent signal fidelity with little noise in the ViT-GAN-SVM model. This indicates that the proposed GAN produces extremely effective stego DWI prostate images while maintaining the signal and structural integrity necessary for medical diagnosis. From the experimental results, it is observed that our ViT-GAN-SVM framework achieves the best performance with 99.78% accuracy, 98.99% precision, 99.85% sensitivity, and finally 99.85% F1-Score. When it comes to prostate cancer identification utilizing DWI pictures, the suggested framework performs noticeably better than current approaches. It is observed that the ViT-GAN-SVM exhibits notable gains in steganographic quality metrics (3.16% to 9.27%) and diagnostic metrics (5.55% to 6.36%).

The ViT-GAN-SVM framework is perfect for cloud-based AI diagnostics (privacy-preserving inference), telepathology (secure remote biopsy review), clinical trials (tamper-proof data sharing), and personalized medicine (protected integration of imaging and genomics), allowing private, verifiable, and diagnostically reliable data exchange across international healthcare systems.

## Data Availability

The data used and/or analyzed during the current study are available from the corresponding author on reasonable request.
